# A mobile system for quantifying the spatial variability of the surface energy balance: design and application

**DOI:** 10.1007/s00484-014-0875-8

**Published:** 2014-07-26

**Authors:** Georg Wohlfahrt, Erich Tasser

**Affiliations:** 1 Institute of Ecology, University of Innsbruck, Sternwartestr. 15, 6020 Innsbruck, Austria; 2 Alpine Environment, European Academy of Bolzano, Drususallee 1, 39100 Bolzano, Italy

**Keywords:** Net radiation, Albedo, Evapotranspiration, Grassland, Land use, Topography

## Abstract

We present a mobile device for the quantification of the small-scale (a few square meters) spatial variability in the surface energy balance components and several auxiliary variables of short-statured (<1 m) canopies. The key element of the mobile device is a handheld four-component net radiometer for the quantification of net radiation, albedo and infrared surface temperature, which is complemented with measurements of air temperature, wind speed, soil temperature and soil water content. Data are acquired by a battery-powered data logger, which is mounted on a backpack together with the auxiliary sensors. The proposed device was developed to bridge between the spatial scales of satellite/airborne remote sensing and fixed, stationary tower-based measurements with an emphasis on micrometeorological, catchment hydrological and landscape–ecological research questions. The potential of the new device is demonstrated through four selected case studies, which cover the issues of net radiation heterogeneity within the footprint of eddy covariance flux measurements due to (1) land use and (2) slope and aspect of the underlying surface, (3) controls on landscape-scale variability in soil temperature and albedo and (4) the estimation of evapotranspiration based exclusively on measurements with the mobile device.

**Electronic supplementary material:**

The online version of this article (doi:10.1007/s00484-014-0875-8) contains supplementary material, which is available to authorized users.

## Introduction

Based on the first law of thermodynamics, the energy balance, Eq. 1, states that the net radiation (*R*_n_) available to a patch of land surface is consumed in the exchange of latent (λ*E*) and sensible (*H*) heat with the atmosphere and the change of heat storage within the system (*S*):

1 $$ {\mathit{\mathsf{R}}}_{\mathsf{n}}=\lambda \mathit{\mathsf{E}}+\mathit{\mathsf{H}}+\mathit{\mathsf{S}} $$

*R*_n_ depends on the net difference between down- (↓) and up-welling (↑) short- (*S*) and long-wave (*L*) radiation, i.e.

2 $$ {\mathit{\mathsf{R}}}_{\mathsf{n}}=\downarrow \mathit{\mathsf{S}}-\uparrow \mathit{\mathsf{S}}+\downarrow \mathit{\mathsf{L}}-\uparrow \mathit{\mathsf{L}} $$

A key component of *R*_n_ is the ratio of up-welling to down-welling shortwave radiation termed albedo (*α*):

3 $$ \alpha =\uparrow \mathit{\mathsf{S}}/\downarrow \mathit{\mathsf{S}} $$

Different types of land surfaces differ in their *R*_n_ which, through Eq. 1, determines how much energy is available for λ*E*, *H* and *S*, which in turn critically affects the near-surface climate (e.g. Stegehuis et al. 2013; Seneviratne et al. 2006). For example, it was shown by Bonan (2008) and Bala et al. (2007) that grasslands and croplands, as opposed to forests, have a cooling effect at higher latitudes because the albedo of grasslands and croplands is typically higher, in particular when covered by snow, compared with forests, which absorb more solar energy. In contrast, in tropical regions, the difference in albedo between forests and grasslands is compensated by the cooling through the large amount of water transpired by (tropical) forests. In order to understand how past (e.g. Brovkin et al. 2006) and potential future (e.g. Bala et al. 2007; Brovkin et al. 2009) changes in land use affect the Earth’s climate, it is crucial to understand how changes in land surface properties affect *R*_n_ and the partitioning into λ*E*, *H* and *S*. For example, it was shown by Chapin et al. (2005) that warming-induced shorter periods of snow cover in the Arctic and associated trends of shrub/tree expansion are likely to cause local warming similar in magnitude to the warming expected from a doubling of atmospheric carbon dioxide concentrations.

The surface energy balance and its components can be quantified by a hierarchy of methods across spatial scales: At the largest scale, merging several satellite data streams with models allows estimating all four components of the energy balance (e.g. Diak et al. 2004; Kalma et al. 2008; Glenn et al. 2007) on a global scale. At the scale of catchments, evapotranspiration may be deduced on an annual basis by difference between precipitation and discharge (e.g. Peel et al. 2010). At the ecosystem-scale, i.e. typically a few hectares characterised by similar vegetation and soil, micrometeorological methods, such as the eddy covariance technique (Baldocchi et al. 1988; Aubinet et al. 2000), allow the direct quantification of both *H* and *λE*, with *R*_n_ and *S* typically being estimated on/from the tower which supports the turbulence equipment (fast-response sonic anemometer and hygrometer). Within the FLUXNET network, the four terms of Eq. 1 are presently measured continuously at >400 sites globally (Baldocchi et al. 2001; Williams et al. 2012). Finally, at the plot, single plant and leaf scale, sap flux (Wilson et al. 2001), various types of chambers and lysimeters (Wohlfahrt et al. 2010a) can be used to quantify (evapo)transpiration.

In this comprehensive hierarchy of methods, it is the lower end of the microscale (Orlanski 1975), that is, spatial variability at the scale of square meters, which is presently poorly represented (e.g. Ahrends et al. 2012). Landscape variability at this spatial scale is much smaller than the typical pixel size of remote sensing-based approaches and also considerably smaller than the typical footprint of micrometeorological measurements. The only approaches suited for this spatial scale, lysimeters and ecosystem chambers, on the other hand, are generally impractical for surveying a large number of distributed samples within the footprint of eddy covariance flux measurements or in a landscape context.

We thus argue that, in micrometeorological, catchment hydrological and landscape ecological studies, there is the need for the development of approaches for spatially distributed energy balance measurements which can be applied at the lower end of the microscale and yet are portable enough to allow making a large number of spatially distributed measurements within short periods of time. To this end, we propose a mobile device which allows quantifying the small-scale (a few square meters) spatial heterogeneity of the energy balance over short-statured (<1 m) canopies. In the following, we first present the design of the mobile device, followed by four case studies which are meant to illustrate its potential and conclude with a discussion of its strengths and weaknesses, as well as an outlook on potential future developments.

## Material and methods

**Fig. 1:**
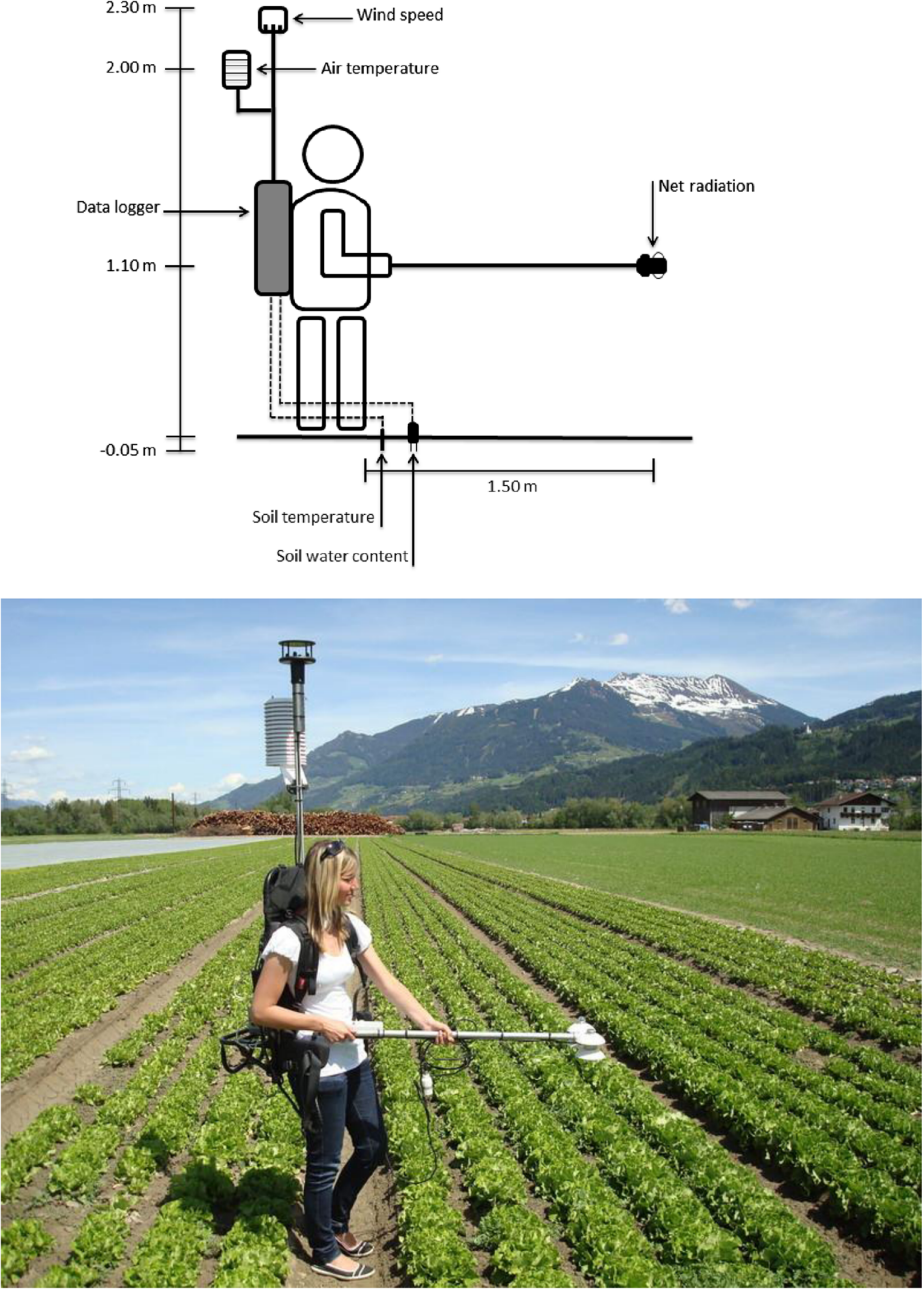
Schematic of the EcoBot design (*top*) and picture showing its application in the field (*bottom*). Indicated heights aboveground in the sketch refer to an operator body size of ca. 1.8 m. See text for further details

The mobile device, referred to as EcoBot, consists of a four-component net radiometer (NR01, Hukseflux, The Netherlands) mounted on a handheld boom, an air temperature/humidity sensor (HMP45C, Campbell Scientific, UK) in a ventilated radiation shield, a two-dimensional sonic anemometer (Windsonic, Gill, UK), a soil temperature (107, Campbell Scientific, UK) and volumetric water content (SM300, Delta-T, UK) sensor, a data logger (CR1000, Campbell Scientific, UK) and a rechargeable battery (12 V, 10 Ah). The data logger and the battery are mounted on a backpack consisting of an aluminium frame, which also supports the radiation shield with the air temperature/humidity sensor and the sonic anemometer on a detachable vertical pole (Fig. 1). The length of the vertical pole may be adjusted to the body size of the operator to result in air temperature and wind speed measurements being made 2.0 and 2.3 m above the ground, respectively. The total weight of the backpack including all sensors is ca. 15 kg. The pole to which the net radiometer is attached features a bubble level for levelling the instrument, as well as push button for triggering measurements. The height above ground of the net radiometer depends somewhat on the body size of the operator, but 1.0–1.1 m above ground have been found to be practical in most cases (Fig. 1), which limits the maximum canopy height to around 1 m. The operator makes measurements with the pole pointing towards South (in the Northern hemisphere), in order to avoid shading of the net radiometer. Due to the field-of-view of the net radiometer (180° and 150° for the pyranometers and pyrgeometers, respectively), it is unavoidable that the operator, similar to supporting structures in a fixed-point setup, affects the radiation measurements. Given the directional response of the net radiometer, this influence is however deemed negligible. In order to reduce variability with different EcoBot operators (e.g. due to differing clothing color), a field stop might be added to the net radiometer for shielding the operator in the future. The sonic anemometer is mounted such that the North arrow points towards North when measurements are made towards South, so that, in principle, the wind direction output may also be used; however, the uncertainty of wind direction (nominal 3°) is estimated to increase to at least 10° due to variability in the position of the operator with respect to true North. The soil temperature and moisture sensors are carefully pushed into the soil down to a depth of ca. 0.05 m (Fig. 1). The data logger is programmed to scan through the program every 5 s. Once the push button is pressed, a digital channel is short-circuited and triggers the data logger to turn on the powered sensors (air temperature and humidity sensor, soil moisture probe, sonic anemometer), acquire a measurement from all sensors and save the current data to the memory, followed, after ca. 2 s, by a sound indicating a successful measurement (see data logger program in the Electronic supplementary material). In stand-by, the EcoBot consumes 55 mA (<1 mA with the ventilation turned off), which increases to 110 mA during the 2-s period when a measurement is taken, allowing 80 and 160 h of continuous measurements and standby with the chosen battery capacity (imposing a residual capacity of 10 %), respectively.

In addition to the four components of *R*_n_ (up- and down-welling short- and longwave radiation; W m^–2^), the data logger outputs the net radiometer body and infrared surface temperature (°C), air and soil temperature (°C), soil moisture (% volumetric soil moisture based on general calibration for mineral soil and raw mV output), wind speed (m s^–1^) and direction (°) as well as a digital sonic data quality flag.

The protocol at each measurement point is the following: First, soil temperature and moisture sensors are put into place. Then the operator gets into position by pointing the net radiometer horizontally (or slope-parallel; see below) towards South and waits 2 min before taking three (pseudo-)replicate measurements at the same spot. The 2-min delay accounts for the time response of the various sensors. The soil moisture sensor and the sonic anemometer have no quoted time response, while the air temperature/humidity sensor and the net radiometer have a quoted time response of <20 s (90 % response). The soil temperature sensor has a quoted response time of <80 s in air at a wind speed of 1 m s^−1^ (63 % response), no indications are given for response times in soil, which are likely to be longer. Following data acquisition, time and place of the measurement and environmental conditions (e.g. cloud cover) are noted in a field book, and any additional measurements are made on the plot (see case study 3 below for an example).

## Results and discussion

In the following, we illustrate the potential of the EcoBot by reference to four selected case studies:

### Case study 1: within eddy covariance footprint heterogeneity of *R*_n_

**Fig. 2:**
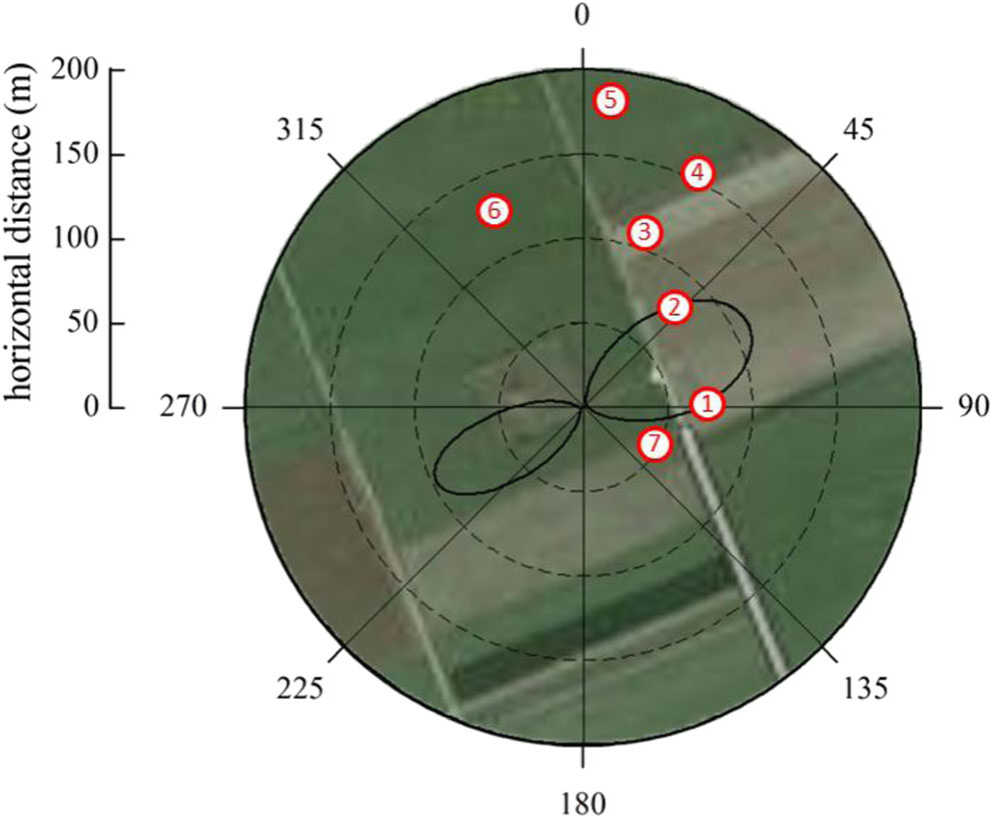
Layout of EcoBot measurements with respect to the eddy covariance flux footprint in Case study 1. EcoBot measurements were made on plots 1–7 referring to the following land uses: (*1*) freshly seeded grassland with a large fraction of visible light brown dry soil, (*2*) densely planted butterhead lettuce (*Lactuca sativa*), (*3*) butterhead lettuce covered with white fleece, (*4*) sparsely planted butterhead lettuce, (*5*) lamb’s lettuce (*Valerianella locusta*), (*6*) grassland similar to stationary tower surrounding, (*7*) grass-dominated grassland. Eddy covariance flux footprints (*solid lines*) enclose the area which contributes more than 1 % of the footprint maximum to the total flux footprint. Flux footprints were calculated with the model by Detto et al. (2006) and refer to a morning situation (06:00–06:30 UTC) with near-neutral stratification and moderate down-valley winds (average horizontal wind speed 2.3 m s^−1^) from Southwest and a situation in the early afternoon (14:30–15:00 UTC) with near-neutral stratification and light up-valley winds (average horizontal wind speed 1.2 m s^−1^) from Northeast

**Fig. 3:**
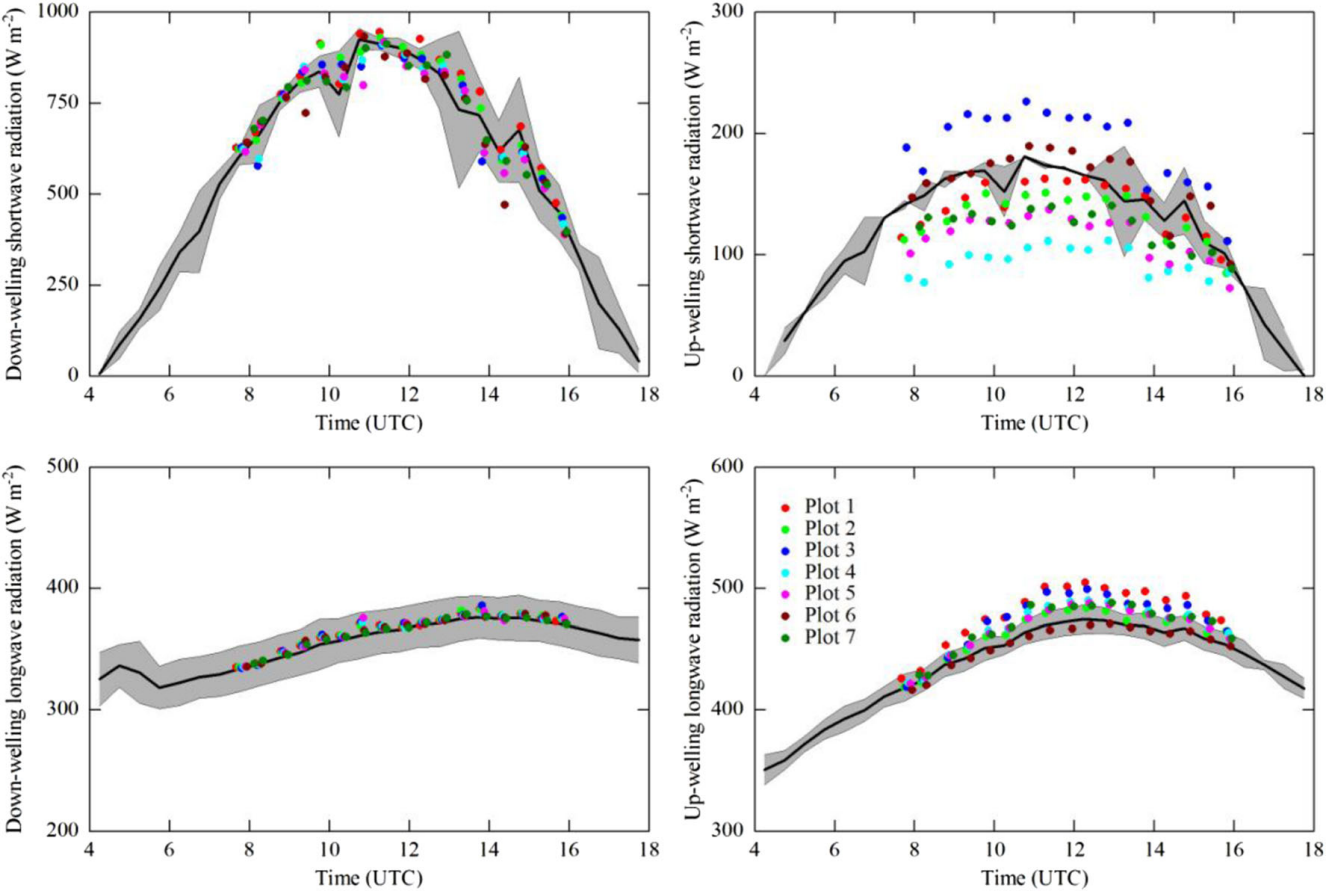
Comparison between stationary (*solid lines with grey shading* indicating 2 × standard deviation) and mobile measurements (at seven different positions characterised by different land use within the eddy covariance flux footprint; see Fig. 2) of the four components of net radiation

In eddy covariance energy flux studies, *R*_n_ and *S* are typically measured either on the tower which supports the turbulence equipment or a nearby additional tower, and in the vast majority of cases, measurements are made at a single location only. As the footprint of eddy covariance flux measurements is typically orders of magnitude larger than the footprint of *R*_n_ and *S* (Schmid 1997), any analyses of *H* and λ*E* that make use of single-point *R*_n_ and *S* rely on the implicit assumption of their values in the flux footprint being equal those measured on the tower. Case study 1, shown in Figs. 2 and 3, was selected as an example illustrating a situation where the tower-based measurements of *R*_n_ differ from *R*_n_ in the footprint due to spatial heterogeneity in vegetation cover caused by land use. Briefly, eddy covariance *H* and λ*E*, *R*_n_ and soil heat flux (*G*; assuming other heat storage to be negligible) measurements were made from a 2-m tower above grassland ca. 20 km to the East of Innsbruck (Austria). The site was situated in the middle of the flat Inn Valley in an area dominated by intensively used grasslands interspersed with various crops (mostly vegetables; Fig. 2). In order to explore the within footprint heterogeneity of *R*_n_, mobile measurements with the EcoBot were conducted on a sunny day (10 May 2012) at the seven dominant land use types within the eddy covariance footprint. To this end, one representative plot was selected within each of the seven land use types and revisited every 30 min and between 8 and 16 UTC and three pseudo-replicate EcoBot measurements made. Figure 3 shows that mobile down-welling shortwave and longwave radiation agreed with the flux tower to within their temporal variability (data from the flux tower were saved as averages and standard deviations over 30 min). In contrast, up-welling shortwave (and thus albedo) and longwave (and thus infrared surface temperature) radiation differed by up to 85 and 35 W m^−2^, respectively, between the seven major land-use types and the stationary measurements (Fig. 3). Due to compensating effects between larger/smaller up-welling radiation fluxes, *R*_n_ at individual plots differed from the stationary measurements at the flux tower by up to ±60 W m^−2^. Depending on the aerial extent of the various land surface types and their contribution to the flux footprint (Fig. 2), these differences may need to be accounted for when relating *R*_n_ to latent and sensible energy fluxes or when attempting to close the energy balance (Foken 2008). Doing so will require a two-dimensional footprint model (e.g. Detto et al. 2006; Kljun et al. 2002), which allows weighting *R*_n_ of the various land use types with their flux contribution.

### Case study 2: estimating slope-parallel *R*_n_

Measurements of *R*_n_ are typically made horizontally, assuming a horizontal underlying surface. However, in case of measurements above sloping terrain, slope-parallel measurements are required to be able to relate *R*_n_ to latent and sensible heat fluxes (Whiteman et al. 1989). Algorithms for correcting horizontal *R*_n_ measurements for slope and aspect of the underlying non-horizontal surface exist, but, however, usually account only for differences between the angle of incident direct solar radiation and the surface (e.g. Matzinger et al. 2003) and, similar to case study 1, do not account for heterogeneity in slope and aspect within the flux footprint (but see Hammerle et al. 2007).

**Fig. 4:**
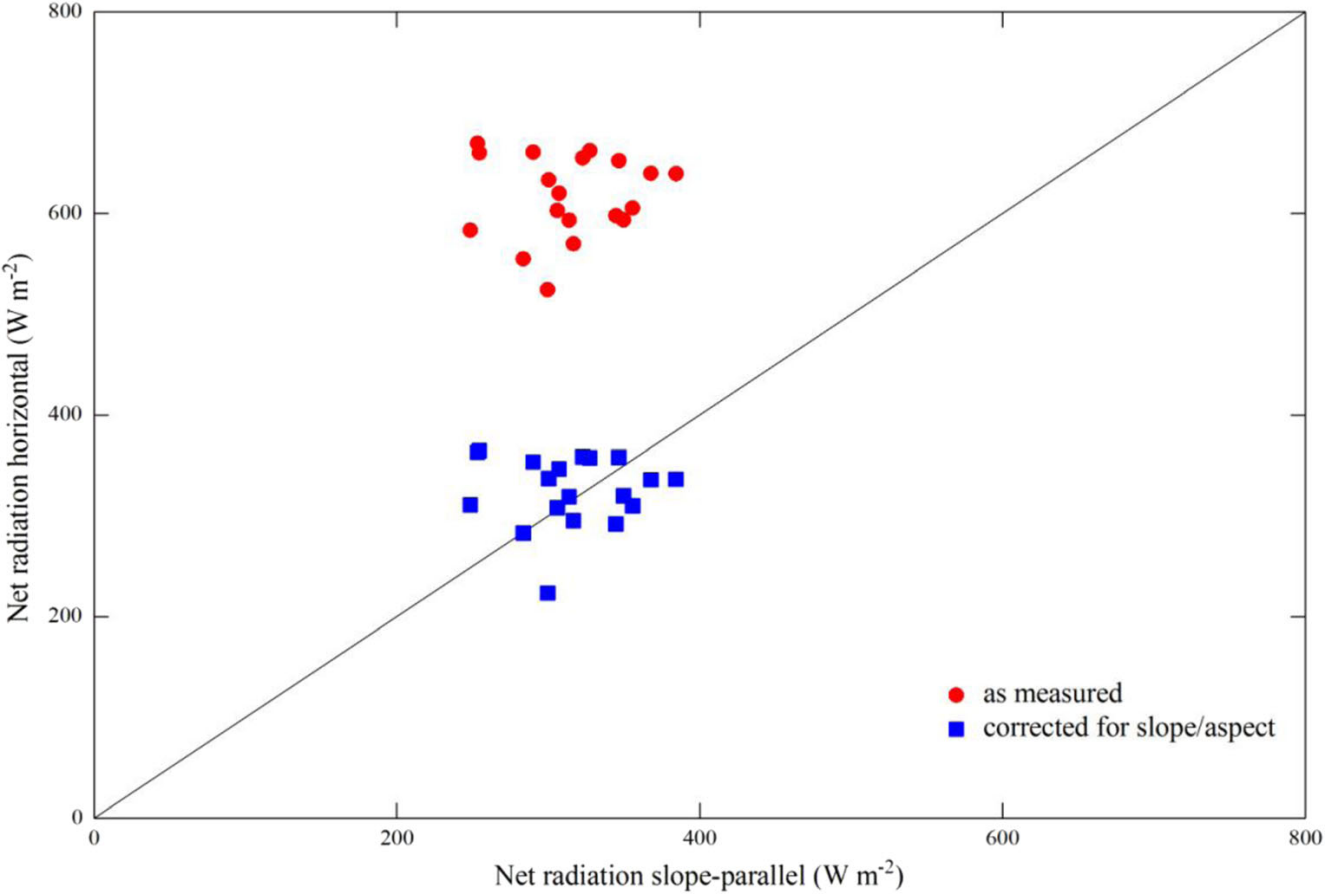
Comparison between net radiation measured parallel to the slope and measured horizontally (*red circles*) and horizontal measurements corrected for local slope and aspect (*blue squares*) (Hammerle et al. 2007)

In case study 2, the EcoBot was used to investigate differences between horizontally and slope-parallel measured *R*_n_ and to quantify the reliability of approaches to correct for slope and aspect. The study site was again ca. 20 km to the East of Innsbruck (Austria), but this time on a grassland site situated high up on a North facing slope with an average inclination of 30°. EcoBot measurements were made on a clear day (14 May 2013) for three times during the day (morning, noontime, afternoon) at seven positions around the flux tower characterised by different slopes and expositions. Two measurements, each with three pseudo-replicates, were made at each plot—the first one horizontally and a second one with the net radiometer approximately inclined according to local slope and aspect based on a manual assessment of the operator. The fraction of diffuse radiation was quantified continuously with quantum sensor (BF3H, Delta-T, UK) on the flux tower. As shown in Fig. 4, it is obvious that horizontal measurements overestimated *R*_n_ measured slope-parallel on this steep North-facing slope by a factor of almost 2. Correcting for local slope and aspect following Hammerle et al. (2007) reduced the discrepancy to the slope-parallel measurements (on average down to 3 %; Fig. 4); however, from the spread of data (differences up to 100 W m^−2^), it is clear that the correction did not completely remove the bias at all locations and times. Apparently, there are other local factors, such as the fraction of cold sky/warm vegetation seen by the pyrgeometers, which vary within the footprint and are not well captured by the common approach of correcting only for the angle between the incident direct short-wave radiation and the underlying surface. In addition, the manual assessment of local slope and aspect is likely to introduce uncertainty, which might be reduced by adding an electronic tilt sensor to the EcoBot capabilities in the future.

### Case study 3: drivers of landscape-scale variability in soil temperature and albedo

Case study 3 is meant to illustrate the potential of EcoBot and concurrent auxiliary measurements to study landscape-scale variability in *R*_n_ and its components and drivers. Briefly, the study was conducted between June and October 2011 and June 2013 in the Stubai Valley (Western Austria), in the Matscher/Mazia Valley and in the Tauferer-Ahrntal Valley (both in Northern Italy), at 51 different grassland and shrub ecosystems. The study sites covered an altitudinal range from 850 to 2,500 m asl and included abandoned areas and differently managed hay meadows and pastures. At each site, two to five replicate EcoBot measurements were taken as described above. At the same sites, the above-ground plant area index (PAI) was estimated directly based on harvesting and plant area determination and/or indirectly based on canopy light transmission measurements using a line quantum sensor as described in Wohlfahrt et al. (2001). The total above-ground biomass was quantified by harvesting the vegetation in a 0.3 × 0.3 m area. Species composition and dominance were estimated in a 2 × 2 m area based on Braun-Blanquet (1964) and the vegetation association according to Tasser et al. (2010).

**Fig. 5:**
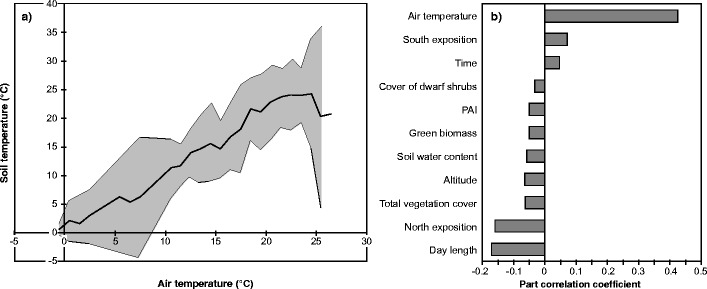
**a** Comparison between air and soil temperature (*solid lines with grey shading* indicating 2 × standard deviation) at different grassland ecosystems. **b** Result of the stepwise linear regression to model the dependence of soil temperature on explanatory variables: The figure shows the part correlation coefficients of the significant explanatory variables

Soil temperature affects numerous soil processes (e.g. weathering, mineralisation of organic material) and through the biogeochemical cycling of carbon, nutrients and water, vegetation composition and status. Spatial differences in soil temperature on a landscape-scale reflect these differences in soil and vegetation, in addition to topographical and environmental factors. Figure 5 illustrates the potential of the EcoBot to explore and explain landscape-scale spatial patterns in soil temperature using a forward stepwise linear regression. We hypothesised that a combination of site, vegetation and land-use variables would best predict spatial soil temperature patterns. The following site variables were used: altitude (as proxy for the altitudinal climate gradient), slope angle and aspect, all parameters measured by the EcoBot (see Fig. 1), day length, time of day, total vegetation cover, cover of grasses, forbs, dwarf shrubs and open soil, mean canopy height, PAI and phytomass (total, green, woody and dead plant matter). With these independent variables, a total of 83.7 % of the spatial variability in soil temperature could be explained (Durbin Watson: 1.4), with 11 variables contributing significantly (Fig. 5b). Spatial patterns of soil temperature were positively correlated with air temperature, which explained the largest fraction of the total variability (Fig. 5a), the time of day and the degree of South exposition. Negative correlations were observed with variables expressing the amount and cover of above-ground plant area (total vegetation and dwarf shrub cover, PAI and green biomass), soil moisture, altitude, day length and the degree of North exposition. While this simple empirical model is likely to have little utility outside the conditions under which the data have been acquired, it may be used in the study areas, together with land cover/use maps and information on the litter quality of single different land cover types (e.g. Gamper et al. 2007), to predict soil mineralisation rates for carbon budgeting studies (e.g. Smith et al. 2005).

**Fig. 6:**
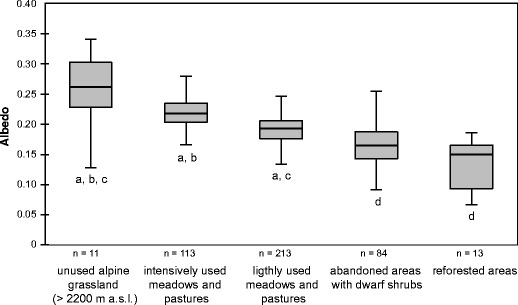
Relationship between land use and albedo. The *boxplots* show median, interquartile range and outliers. Group differences were tested with a Dunnett-T3 test (*p* < 0.05). The letters (*a*, *b*, *c*, *d*) refer to significant differences between the land use-types

The potential of the EcoBot for exploring spatial differences in albedo driven by land use is shown in Fig. 6 for the same dataset. Abandoned areas with a high cover of dwarf shrubs or taller woody shrub species such as *Pinus mugo* or *Alnus virridis* reflected much less shortwave radiation compared with differently managed grasslands or Alpine grasslands above the tree line (Fig. 6). These findings are critical for accounting for biophysical feedbacks (Bonan 2008) from ongoing changes in land use (e.g. Tappeiner et al. 2008a, b) and climate (e.g. Pauli et al. 2007; Gobiet et al. 2014) in the Alps.

### Case study 4: using the EcoBot for inferring evapotranspiration

Another application, which primarily motivated the inclusion of air temperature and wind speed measurements, is to use the EcoBot for estimating sensible and latent heat fluxes. In a bulk approach, the sensible heat flux may be derived from the gradient (K) between the aerodynamic surface temperature (*T*_aero_) and air temperature (*T*_air_), divided by the aerodynamic resistance to heat transfer (*r*_aero_; s m^−2^) multiplied by the product of the density and specific heat of air (*ρc*_*p*_; J m^−3^ K^−1^):

4 $$ \mathit{\mathsf{H}}=\rho {\mathit{\mathsf{c}}}_{\mathit{\mathsf{p}}}\left({\mathit{\mathsf{T}}}_{\mathsf{aero}}-{\mathit{\mathsf{T}}}_{\mathsf{air}}\right)/{\mathit{\mathsf{r}}}_{\mathsf{aero}} $$

For near-neutral conditions, the aerodynamic resistance may be estimated on the basis of the logarithmic wind law using measured wind speed and estimates of the zero-plane displacement height (m) and roughness length (m), which may be derived from canopy height (Campbell and Norman 1998). If the aerodynamic surface temperature is replaced with the measured infrared surface temperature, Eq. 4 may be solved for *H* exclusively on the basis of EcoBot measurements. Replacing the heat storage (*S*) in Eq. 1 with the soil heat flux (*G*) and assuming *G* to represent some fraction of *R*_n_ (Sauer and Horton 2005) and/or by empirically relating it to measured soil temperature and water content, *H* derived from Eq. 4 together with estimated *G* and measured *R*_n_ allows inferring λ*E* as the residual of the energy balance, i.e.

5 $$ \lambda \mathit{\mathsf{E}}={\mathit{\mathsf{R}}}_{\mathsf{n}}-\mathit{\mathsf{H}}-\mathit{\mathsf{G}} $$

**Fig. 7:**
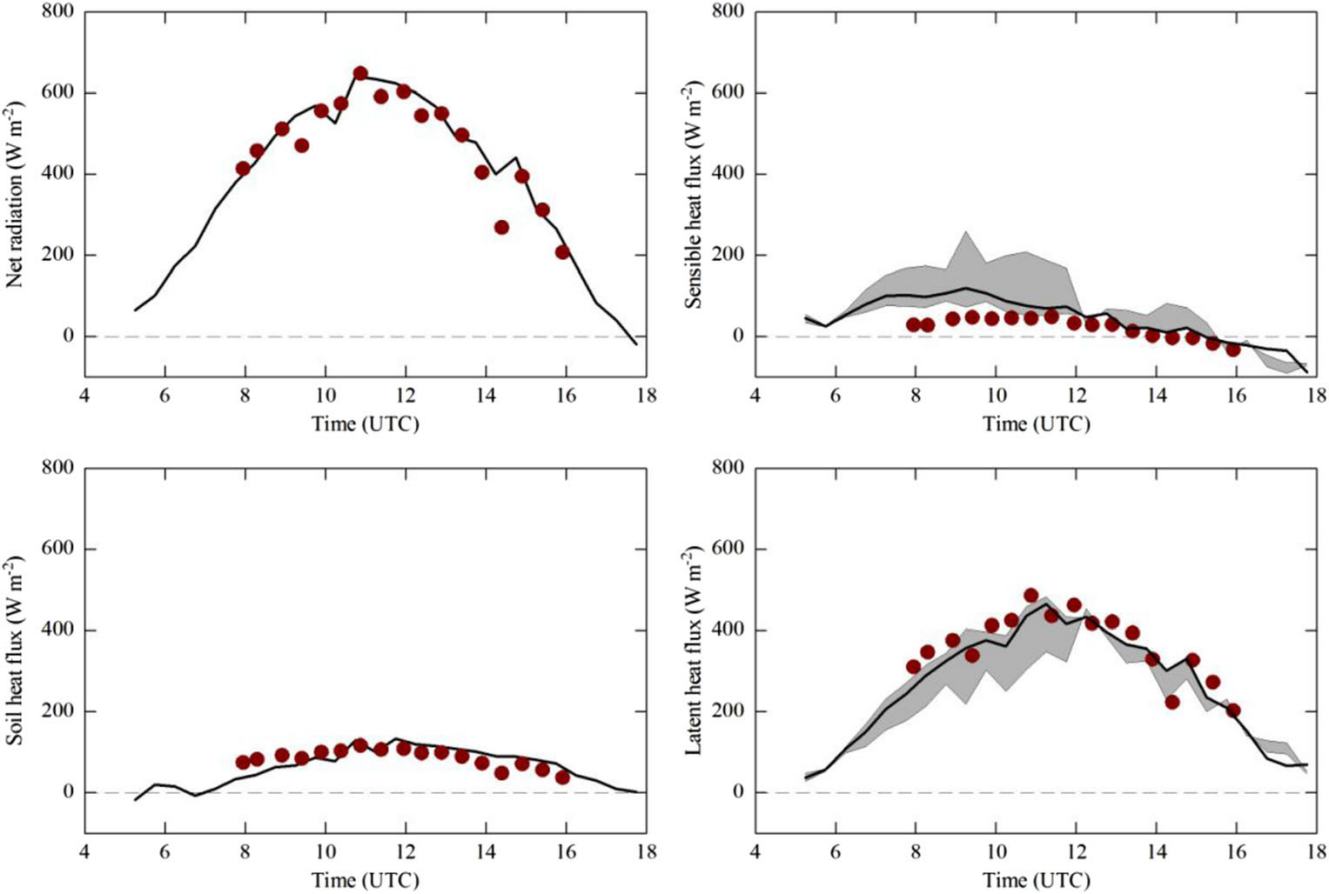
Energy flux components as measured on an eddy covariance flux tower (*lines*) and measured/estimated based on the data from the EcoBot (*symbols*; corresponding to plot 6 in Figs. 2 and 3). The *solid lines* in the sensible and latent heat flux sub-panels refer to the respective flux with the residual energy distributed according to the Bowen ratio; the *grey areas* refer to the range between the sensible (latent) heat fluxes without closure (lower range) and with the entire energy imbalance allocated to the sensible (latent) heat flux (upper range) (Wohlfahrt et al. 2009). See text for further details

This approach was applied to the data presented in case study 1, and the results are shown in Fig. 7 for the four components of the energy balance equation. Note that, in contrast to the data shown in Fig. 3, here we present EcoBot measurements only from the grassland plot (#6 in Figs. 2 and 3) which is identical to where the eddy covariance flux tower is situated and which makes up a major fraction of the flux footprint. In order to enable comparison with the EcoBot calculations, which close the energy balance by definition (Eq. 5), eddy covariance sensible and latent heat fluxes were adjusted for the lack of energy balance closure (20 % residual energy on average) using three different approaches (Wohlfahrt et al. 2009): The first approach forces closure by assigning the residual energy to *H* and λ*E* according to the Bowen-ratio and is used as the reference (solid lines in Fig. 7) below. The second approach assigns the entire residual energy to either *H* or λ*E*, and the third approach uses *H* and λ*E* as measured, i.e. applies no closure operation. The second and third approaches represent the range of possible closure operations and are highlighted in Fig. 7 by grey shading. The soil heat flux was estimated as 18 % of *R*_n_ measured by the EcoBot, based on soil heat flux measurements at the eddy covariance flux tower (see also Hammerle et al. 2008). It can be seen that, despite a clear underestimation of *H* before noon, overall λ*E* inferred from the Ecobot measurements nicely corresponded with the one measured by eddy covariance (λ*E*_EcoBot_ = 1.05 λ*E*_*EC*_, *r* ^2^ = 0.79, RMSE = 40.3 W m^−2^) and was mostly within the range of the uncertainty of the eddy covariance λ*E* measurements due to the lack of energy balance closure.

Clearly, the assumptions involved in and uncertainties associated with this approach, in particular, the crude estimation of the soil heat flux (Sauer and Horton 2005), the replacement of the aerodynamic surface temperature with the infrared surface temperature (e.g. Kustas and Norman 1996) and the calculation of the aerodynamic resistance to heat transfer (Liu et al. 2007), are likely to be significant. It is well-known that the difference between the aerodynamic and infrared surface temperature (RMSE = 1.9 K for the data shown in Fig. 7) may become substantial in situations with partial canopy cover, necessitating semi-empirical corrections of Eq. 4 (e.g. Kalma et al. 2008). The encouraging results shown in Fig. 7 may thus partially be owed to the relatively ideal circumstances, such as the high leaf area index of ca. 4 m^2^ m^−2^, within which the comparison with the eddy covariance fluxes was conducted. In addition, atmospheric conditions need to be steady and/or appropriate temporal averaging be applied to the EcoBot data for deriving meaningful energy fluxes. With these caveats in mind, we conclude that the estimation of *G*, *H* and λ*E* based solely on EcoBot data requires further testing across a larger number of different ecosystems. At the same time, we stress that the preliminary evidence presented in Fig. 7 suggests the EcoBot to offer exciting potential for estimating the small-scale spatial variability in evapotranspiration in a landscape context, which is difficult to realise with other approaches. For example, the EcoBot may provide critical data for interpreting streamflow data and for the calibration/validation of evapotranspiration simulated by distributed hydrological models (e.g. Rigon et al. 2006) in catchment hydrological studies or for the ground validation of satellite products (Pasolli et al. 2011). In particular for ecosystems where microtopography strongly governs vegetation distribution, such as in Arctic or Alpine ecosystems (e.g. Scherrer and Körner 2011; Gamon et al. 2013), the EcoBot may offer considerable advantage over other approaches.

### Conclusions and outlook

We have presented a mobile device, termed EcoBot, which allows quantifying the small-scale (a few square meters) spatial variability in the surface energy balance, its components (in particular evapotranspiration, net radiation and albedo) and several auxiliary variables (e.g. soil temperature and water content) of short-statured canopies. The proposed device was developed to bridge between the spatial scales of satellite/airborne remote sensing and fixed single-tower net radiation measurements with an emphasis on micrometeorological, catchment hydrological and landscape–ecological research questions. Due to the one-point-in-time nature of the measurements, the EcoBot will be most useful during intensive campaigns when small-scale spatial coverage is more important than long-term measurements. As illustrated in four selected case studies, the proposed device appears to offer potential for the interpretation of within-footprint heterogeneity effects on eddy covariance energy flux measurements (Figs. 2, 3, and 4), for questions related to landscape-scale spatial variability of the surface energy balance, its components and drivers (Figs. 5, 6, and 7) and thus more generally for validation of energy balance satellite products and distributed hydrological models. In particular during satellite/aerial overpasses, the EcoBot may provide an efficient means to acquire, complementary to stationary measurements, spatially distributed ground truth data.

Provided the proposed measurement protocol is followed, the EcoBot offers a reliable approach to measure a larger number of spatially distributed sampling points (possibly with the exception of soil temperature due to the relatively long time constant of the sensor). In combination with additional plant- (e.g. amount and composition of above-ground phytomass) and soil-related (e.g. soil type, colour) parameters, these measurements offer new avenues for research into the role of small-scale spatial variability of vegetation and soil for land–atmosphere coupling. The inferred distribution of *R*_n_ into *G*, *H* and λ*E* represents an even more exciting possible application of the EcoBot; however, due to the assumptions involved, it requires further testing. The preliminary comparison with direct eddy covariance flux measurements presented in Fig. 7 is however encouraging.

The EcoBot was designed for short-statured canopies, less than approximately 1 m tall, which allow a convenient operation of the net radiometer. Using a ladder, we anticipate that it would be possible to use the EcoBot for canopy heights up to around 2 m, such as larger bushes, agricultural crops or young trees. For taller canopies, such as adult forests, airborne measurements are likely to remain the only alternative. The EcoBot may however be used to quantify the spatial variability of *R*_n_ in the forest understory.

The capabilities of the EcoBot may be easily augmented by adding additional sensors. One promising option would be to include a pair of down- and upward looking multi-spectral or photosynthetically active radiation (PAR) sensors. Multi-spectral sensors are available in configurations that allow calculation of frequently used vegetation indices such as photochemical reflectance index (Gamon et al. 1992) or normalised difference vegetation index (NDVI; Tucker 1979). By difference with the up- and down-welling shortwave radiation measurements, PAR sensors allow calculating a so-called broadband NDVI (Huemmrich et al. 1999). Acquisition of these additional data would further strengthen the link between Ecobot data and satellite/airborne remote sensing and provides proxies for the amount of vegetation and its photosynthetic activity (Wohlfahrt et al. 2010b; Huemmrich et al. 1999; Richardson et al. 2007; Eklundh et al. 2011).

## Electronic supplementary material

Below is the link to the electronic supplementary material.

ESM 1 (CR1 5 kb)
